# Supplementary material for: A Plant-Based Meal Stimulates Incretin and Insulin Secretion More Than an Energy- and Macronutrient-Matched Standard Meal in Type 2 Diabetes: A Randomized Crossover Study
Source: Nutrients. 2019 Feb 26;11(3):486. doi: 10.3390/nu11030486 (PMC6471274; doi:10.3390/nu11030486)
Supplement: Supplementary file 1 [file nutrients-11-00486-s001.zip › Suppl Fig. 1 Flowchart dia.pptx]

## Slide 1
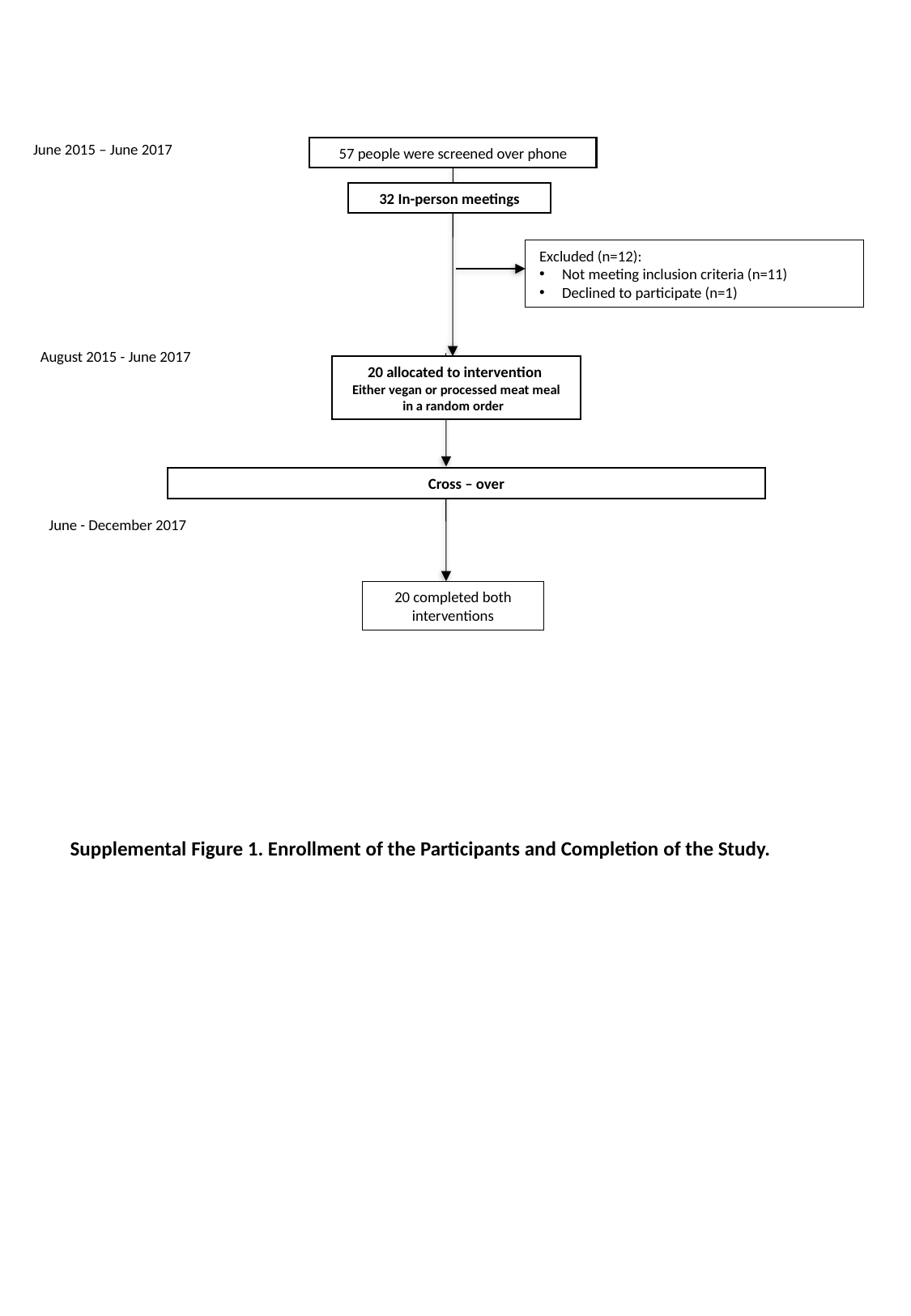

June 2015 – June 2017
57 people were screened over phone
August 2015 - June 2017
June - December 2017
20 completed both interventions
32 In-person meetings
Excluded (n=12):
Not meeting inclusion criteria (n=11)
Declined to participate (n=1)
20 allocated to intervention
Either vegan or processed meat meal in a random order
Cross – over
Supplemental Figure 1. Enrollment of the Participants and Completion of the Study.
