# Supplementary material for: A Plant-Based Meal Stimulates Incretin and Insulin Secretion More Than an Energy- and Macronutrient-Matched Standard Meal in Type 2 Diabetes: A Randomized Crossover Study
Source: Nutrients. 2019 Feb 26;11(3):486. doi: 10.3390/nu11030486 (PMC6471274; doi:10.3390/nu11030486)
Supplement: Supplementary file 1 [file nutrients-11-00486-s001.zip › Suppl. Fig2 Postprandial changes.pptx]

## Slide 1
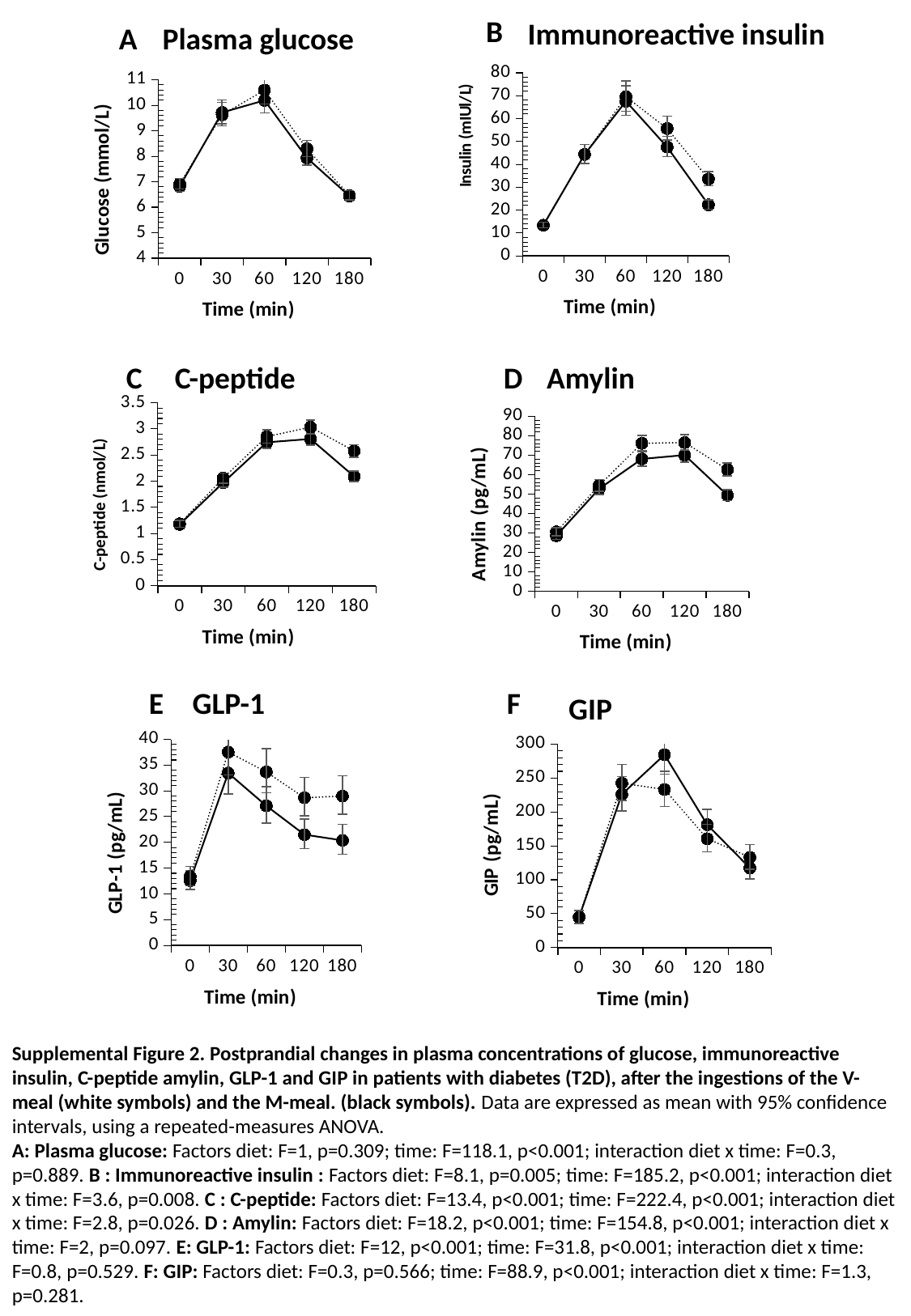

B
Immunoreactive insulin
Plasma glucose
A
### Chart
| Category | | |
|---|---|---|
| 0 | 13.423597513002878 | 13.4081680772025 |
| 30 | 44.37122960614688 | 44.33818441930519 |
| 60 | 67.58466400068792 | 69.55326060413064 |
| 120 | 47.54799057292867 | 55.63750910410249 |
| 180 | 22.320416655999335 | 33.65503548121184 |
### Chart
| Category | | |
|---|---|---|
| 0 | 6.817357026087381 | 6.912755050333251 |
| 30 | 9.71680435943313 | 9.623755179828532 |
| 60 | 10.190793475633232 | 10.592598486605548 |
| 120 | 7.930497519889032 | 8.29307960266256 |
| 180 | 6.437644966231865 | 6.4619576973840935 |Amylin
C-peptide
D
C
### Chart
| Category | | |
|---|---|---|
| 0 | 1.1742722669209913 | 1.18531823692468 |
| 30 | 1.9730292726998775 | 2.0605387753377267 |
| 60 | 2.743580903214111 | 2.852358772879377 |
| 120 | 2.807316601656078 | 3.0344815895083666 |
| 180 | 2.0913493448359173 | 2.577571589853481 |
### Chart
| Category | | |
|---|---|---|
| 0 | 28.579691733555304 | 30.596477649057743 |
| 30 | 52.74988830696989 | 54.451939740855224 |
| 60 | 68.10718130396927 | 76.14873456600839 |
| 120 | 70.10926397984305 | 76.55201243639176 |
| 180 | 49.40452576918 | 62.602220052470344 |GLP-1
F
E
GIP
### Chart
| Category | | |
|---|---|---|
| 0 | 12.551053070782457 | 13.349850973761145 |
| 30 | 33.45347002704282 | 37.5322947798718 |
| 60 | 27.096968943175114 | 33.667451300042266 |
| 120 | 21.50241874558327 | 28.65988612287303 |
| 180 | 20.384625679854867 | 28.98629223344427 |
### Chart
| Category | | |
|---|---|---|
| 0 | 44.93732933719561 | 44.58134192590558 |
| 30 | 225.8709398178744 | 242.5032011779875 |
| 60 | 284.4860967424661 | 233.1562357738947 |
| 120 | 181.15746994232364 | 160.58803008289377 |
| 180 | 117.2688642343779 | 133.18560977672485 |Supplemental Figure 2. Postprandial changes in plasma concentrations of glucose, immunoreactive insulin, C-peptide amylin, GLP-1 and GIP in patients with diabetes (T2D), after the ingestions of the V-meal (white symbols) and the M-meal. (black symbols). Data are expressed as mean with 95% confidence intervals, using a repeated-measures ANOVA.
A: Plasma glucose: Factors diet: F=1, p=0.309; time: F=118.1, p<0.001; interaction diet x time: F=0.3, p=0.889. B : Immunoreactive insulin : Factors diet: F=8.1, p=0.005; time: F=185.2, p<0.001; interaction diet x time: F=3.6, p=0.008. C : C-peptide: Factors diet: F=13.4, p<0.001; time: F=222.4, p<0.001; interaction diet x time: F=2.8, p=0.026. D : Amylin: Factors diet: F=18.2, p<0.001; time: F=154.8, p<0.001; interaction diet x time: F=2, p=0.097. E: GLP-1: Factors diet: F=12, p<0.001; time: F=31.8, p<0.001; interaction diet x time: F=0.8, p=0.529. F: GIP: Factors diet: F=0.3, p=0.566; time: F=88.9, p<0.001; interaction diet x time: F=1.3, p=0.281.
