# Supplementary material for: A Plant-Based Meal Stimulates Incretin and Insulin Secretion More Than an Energy- and Macronutrient-Matched Standard Meal in Type 2 Diabetes: A Randomized Crossover Study
Source: Nutrients. 2019 Feb 26;11(3):486. doi: 10.3390/nu11030486 (PMC6471274; doi:10.3390/nu11030486)
Supplement: Supplementary file 1 [file nutrients-11-00486-s001.zip › Suppl Fig 3 Correlations dia.pptx]

## Slide 1
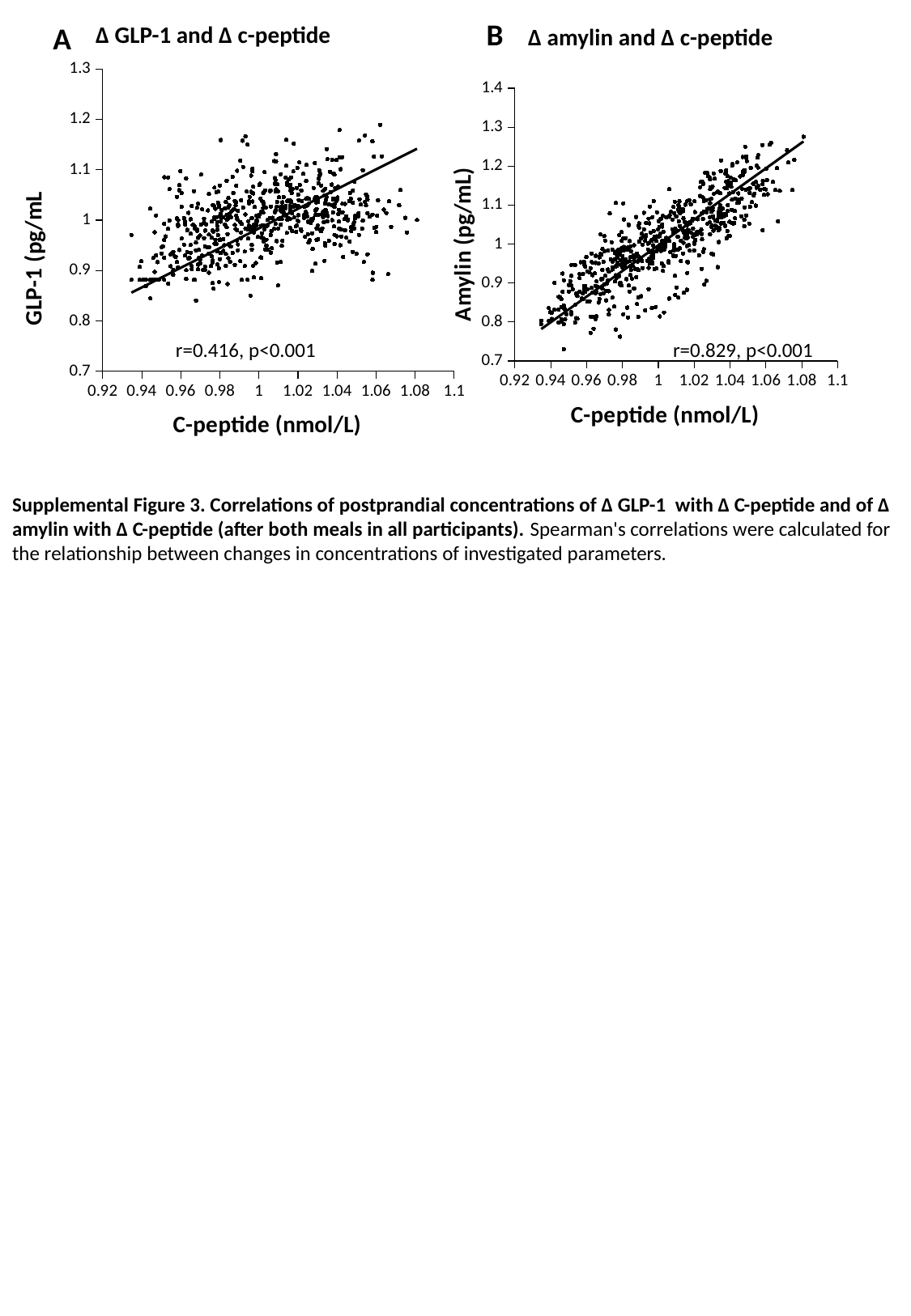

B
Δ GLP-1 and Δ c-peptide
A
Δ amylin and Δ c-peptide
### Chart
| Category | | |
|---|---|---|
### Chart
| Category | | |
|---|---|---|r=0.416, p<0.001
r=0.829, p<0.001
Supplemental Figure 3. Correlations of postprandial concentrations of Δ GLP-1 with Δ C-peptide and of Δ amylin with Δ C-peptide (after both meals in all participants). Spearman's correlations were calculated for the relationship between changes in concentrations of investigated parameters.
